# Reframing the concept of alternative livelihoods

**DOI:** 10.1111/cobi.12607

**Published:** 2015-11-02

**Authors:** Juliet H. Wright, Nicholas A. O. Hill, Dilys Roe, J. Marcus Rowcliffe, Noëlle F. Kümpel, Mike Day, Francesca Booker, E. J. Milner‐Gulland

**Affiliations:** ^1^ Imperial College Conservation Science, Department of Life Sciences, Imperial College LondonSilwood Park CampusBuckhurst RoadAscotSL5 7PYUnited Kingdom; ^2^ Zoological Society of LondonRegent's ParkLondonNW1 4RYUnited Kingdom; ^3^ Conservation Science Group, Department of ZoologyUniversity of CambridgeDowning Street, CambridgeCB2 3EJUnited Kingdom; ^4^ International Institute for Environment and Development80‐86 Gray's Inn RoadLondonWC1X 8NHUnited Kingdom; ^5^ Center for International Forestry ResearchJalan CIFORSitu Gede, Sindang BarangBogor16115Indonesia

**Keywords:** complexity, diversification, integrated conservation and development projects, natural resource management, poverty, sustainable livelihoods, complejidad, conservación integrada y proyectos de desarrollo, diversificación, manejo de recursos naturales, pobreza, subsistencias sustentables

## Abstract

Alternative livelihood project (ALP) is a widely used term for interventions that aim to reduce the prevalence of activities deemed to be environmentally damaging by substituting them with lower impact livelihood activities that provide at least equivalent benefits. ALPs are widely implemented in conservation, but in 2012, an International Union for Conservation of Nature resolution called for a critical review of such projects based on concern that their effectiveness was unproven. We focused on the conceptual design of ALPs by considering their underlying assumptions. We placed ALPs within a broad category of livelihood‐focused interventions to better understand their role in conservation and their intended impacts. We dissected 3 flawed assumptions about ALPs based on the notions of substitution, the homogenous community, and impact scalability. Interventions based on flawed assumptions about people's needs, aspirations, and the factors that influence livelihood choice are unlikely to achieve conservation objectives. We therefore recommend use of a sustainable livelihoods approach to understand the role and function of environmentally damaging behaviors within livelihood strategies; differentiate between households in a community that have the greatest environmental impact and those most vulnerable to resource access restrictions to improve intervention targeting; and learn more about the social–ecological system within which household livelihood strategies are embedded. Rather than using livelihood‐focused interventions as a direct behavior‐change tool, it may be more appropriate to focus on either enhancing the existing livelihood strategies of those most vulnerable to conservation‐imposed resource access restrictions or on use of livelihood‐focused interventions that establish a clear link to conservation as a means of building good community relations. However, we recommend that the term ALP be replaced by the broader term livelihood‐focused intervention. This avoids the implicit assumption that alternatives can fully substitute for natural resource‐based livelihood activities.

## Abstract (es)

Reestructuración del Concepto de las Subsistencias Alternativas

**Resumen**

El término proyecto de subsistencia alternativa es utilizado ampliamente para las intervenciones que buscan reducir la prevalencia de las actividades señaladas como dañinas para el ambiente al sustituirlas con actividades de subsistencia de menor impacto que proporcionan por lo menos beneficios equivalente. Estos proyectos se implementan comúnmente en la conservación, pero en 2012, una resolución de la Unión Internacional para la Conservación de la Naturaleza pidió una revisión crítica de dichos proyectos con base en la preocupación por la falta de pruebas de su efectividad. Nos enfocamos en el diseño conceptual de proyectos alternativos de subsistencia al considerar sus conjeturas subyacentes. Colocamos los proyectos alternativos de subsistencia dentro de una categoría amplia de intervenciones enfocadas en la subsistencia para entender de mejor manera su papel en la conservación y sus impactos intencionales. Analizamos minuciosamente tres suposiciones erróneas sobre los proyectos de subsistencia alternativa con base en las ideas de sustitución, comunidad homogénea y escalabilidad del impacto. Las intervenciones basadas en las suposiciones erróneas de las necesidades de las personas, aspiraciones y los factores que influyen en la elección de la subsistencia tienen poca probabilidad de alcanzar objetivos de conservación. Por lo tanto, recomendamos el uso de una estrategia de subsistencias sustentables para entender el papel y la función de los comportamientos dañinos para el ambiente dentro de las estrategias de subsistencia; diferenciar entre los hogares de una comunidad que tienen el mayor impacto ambiental y aquellos más vulnerables a las restricciones de acceso a los recursos para mejorar la selección de intervenciones; y aprender más sobre el sistema socio‐ecológico en el cual están embebidas las estrategias de subsistencia de los hogares. En lugar de usar las intervenciones enfocadas en la subsistencia como una herramienta directa de cambio de comportamiento, puede ser más apropiado enfocarse en mejorar las estrategias existentes de aquellos más vulnerables a las restricciones de acceso a los recursos impuestas por la conservación o en el uso de las intervenciones enfocadas en la subsistencia que establecen un vínculo claro con la conservación como medio de construcción de buenas relaciones comunitarias. Sin embargo, recomendamos que el término proyecto de subsistencia alternativa sea remplazado por el término más general de intervención enfocada en la subsistencia. Esto evita la suposición implícita de que las alternativas pueden sustituir por completo a las actividades de subsistencia basadas en los recursos naturales.

## Introduction

There has been much debate among academics, practitioners, and policy‐makers with regard to the degree to which conservationists should focus on social issues (Roe 2008; Miller et al. 2011). In developing countries, both pragmatic and ethical arguments can be made as to why conservation should address issues such as poverty, human welfare, social justice, livelihood enhancement, and economic development (Robinson 2011). Broad social concerns have been receiving attention from conservation practitioners since the 1980s, when integrated conservation and development projects gained popularity as a win‐win strategy linking biodiversity conservation with the social and economic development of neighboring communities (McShane & Wells 2004). A paradigm shift toward people‐centered conservation in the 1990s resulted in a suite of other approaches aimed at involving local people in conservation, including community‐based conservation, community‐based natural resource management, and integrated coastal zone management. Interventions that aim to change or enhance the livelihoods of local people often form part of these approaches. The so‐called alternative livelihood project (ALP) is one such intervention, which has been implemented in a range of contexts to reduce reliance on natural resources, generate economic benefits, and increase local support for conservation.

Designed to reduce the prevalence of behaviors that are considered environmentally damaging and unsustainable, ALPs promote substitute, or lower impact, livelihood activities. However, the effectiveness of ALPs, and people‐centered conservation approaches in general, has been questioned. Disenchantment began in the mid‐1990s when these approaches were criticized as having minimal, or even adverse, effects on biodiversity conservation (Oates 1995; Noss 1997). One of the few quasi‐experimental studies exploring the causal impacts of ALPs, conducted in the Brazilian Amazon, found no discernible conservation outcomes (Bauch et al. 2014), yet such studies are rare and in general the amount and rigor of outcome monitoring is low (Brooks et al. 2012; Wicander & Coad 2015). Although substantial evidence of the potential for win‐wins is yet to materialize, conservation still needs to engage with local people, so people‐centered conservation approaches continue to evolve and ALPs reappear in different guises (Redford et al. 2013).

The "new conservation" paradigm focuses on the economic value of nature and seeks to engage people in conservation for utilitarian rather than moral or aesthetic reasons (Kareiva 2014). Market‐based incentives, such as payments for ecosystem services (PES), have been advocated as a direct and cost‐effective approach to people‐centered conservation (Ferraro & Kiss 2002). However, problems associated with direct cash payments have resulted in a renewed interest in the provision of indirect and in‐kind incentives based on cooperative and reciprocal arrangements (Clements et al. 2010; Cranford & Mourato 2011). These incentive schemes often share many similarities with ALPs, despite not being branded as such. Therefore, even though there is uncertainty regarding the effectiveness of ALPs, they continue to be a key strategy in both the terrestrial and marine conservation realms, and the sharing of lessons learned remains essential.

At the International Union for Conservation of Nature (IUCN) World Conservation Congress in 2012, a resolution was passed calling for a critical review of ALPs and the development of best practice guidelines to ensure sustainable benefits to species, ecosystems, and people (IUCN 2012). This call has resulted in renewed interest in searching for evidence of the success or failure of ALPs, and a systematic review exploring conservation outcomes is currently underway (Roe et al. 2014). The outcomes associated with any conservation project are the result of a conceptual design as well as an implementation process, but the conceptual designs of ALPs are often based on inaccurate assumptions about the social systems within which they operate. These assumptions may be based on the perceptions and values of managers and policy‐makers removed from local realities (Cundill et al. 2011). In addition, the term *ALP* is ambiguous; the role and function of ALPs within broader conservation strategies are poorly defined. Without clearly defining what a project aims to achieve, it is very difficult to measure its impact (Salafsky et al. 2001).

We first sought to clarify the different types of livelihood‐focused interventions (a broad category of conservation interventions which includes ALPs) in order to better understand their role in conservation and their intended impacts. Then, we examined some of the conceptual shortcomings of ALPs specifically by considering key assumptions made during their design and implementation. Next, we gleaned insights from the livelihoods literature to determine how conservation practitioners' understanding of the social context at their project sites could be improved. This would enable them to design more effective livelihood‐focused interventions. Finally, we critically evaluated the usefulness of the term *ALP* in the light of these insights.

## Types of Livelihood‐Focused Interventions

Livelihood‐focused interventions can be grouped into 3 broad and overlapping categories: alternatives, compensation, and incentives. Alternatives partially or completely substitute for the benefits (monetary and nonmonetary) that would normally be obtained from the exploitation of particular natural resources. The assumption often underlying this approach is that pressure on natural resources is primarily caused by poverty and a lack of options (Brown 2002). Roe et al. (2014) subdivide alternatives into 3 categories: those that provide an alternative resource to the one being exploited, for example promoting imported animal protein as an alternative to locally hunted bushmeat; those that provide an alternative occupation so as to reduce the need to exploit natural resources for income, for example promoting butterfly farming as a substitute for expanding agriculture (Morgan‐Brown et al. 2010); and those that encourage an alternative method of exploiting a resource that has a lower impact than the original method, for example promoting fuel‐efficient stoves to reduce the need to fell trees for firewood (DeWan et al. 2013) or changing marketing strategy to increase incomes from the sale of wild coffee, thus reducing the need to convert more forest into farmland (Lilieholm & Weatherly 2010).

Interventions that provide compensation or incentives may promote very similar alternatives under the banner of in‐kind payments, but the conditions under which these are implemented differ. Compensation schemes involve explicit acknowledgment of the social and individual costs of conservation, particularly with regard to access restrictions that negatively affect local people's livelihoods, and aim to adequately compensate for the losses incurred. Such schemes may be based on the principles of social justice and human rights or they may be implemented as palliative measures specifically to reduce conflict (Springer 2009). In contrast, incentive schemes such as PES only provide alternatives as in‐kind payment if people change their behavior in accordance with agreements negotiated in advance (Wunder 2013). Payments for ecosystem services therefore link the promoted alternatives more directly to conservation objectives. For example, in Cambodia, 2 PES schemes were implemented that could be described as ALPs. One provided alternative occupations through an ecotourism venture, and the other an alternative method of selling rice at a premium price through village‐based associations. Both schemes aimed to enhance household incomes without the need to hunt or convert important bird habitat into agricultural or residential land, but participation was contingent upon adherence to locally agreed no‐hunting rules and land‐use plans (Clements et al. 2010).

## Assumptions Underlying ALPs

Although motivations for and assumptions behind individual projects differ, 3 key assumptions underlie many ALPs. The first assumption is that providing alternatives will reduce people's need and desire to exploit natural resources (Sievanen et al. 2005). If given the choice, it is assumed that individuals dependent on unsustainable practices will decide partially or completely to substitute an environmentally damaging activity for the more environmentally sustainable activity being offered. This can be conceptualized in terms of the alternative making the opportunity cost of the destructive activity higher, assuming that the promoted activity is indeed a more productive use of labor than the original activity or that the individuals concerned have an appreciation of trading short‐term losses for long‐term gain. This refocusing of effort away from unsustainable activities is also assumed to increase household resilience in the long term (Marschke & Berkes 2006). A recent study reviewed 15 ALPs in Central Africa, and showed that 8 had been based on the hypothesis that the alternatives would provide the same or more income than hunting, which would mean hunters no longer needed to hunt (Wicander & Coad 2015). However, the evidence suggests that the assumption of substitution rarely holds; the alternatives instead become supplementary sources of income and exploitation of the resource continues at similar levels (Torell et al. 2010). The additional income may even subsidize higher levels of exploitation by enabling the purchase of more efficient equipment (Damania et al. 2005).

To be a genuine substitute, the promoted alternative must align with the needs and aspirations of the people concerned and fulfill the same range of functions characteristic of the original activity. For instance, as well as providing cash or noncash income, the alternative may need to function as a safety net or offer similar levels of prestige and job satisfaction (Pollnac & Poggie 2008). Hunting for bushmeat, for example, has many positive attributes as a livelihood activity in West and Central Africa. Barriers to entry are low and labor inputs are flexible, making hunting compatible with the agricultural cycle (Brown & Williams 2003). The ability to generate income quickly means hunting also plays an important safety net function during short‐term crises (Schulte‐Herbrüggen et al. 2013). Developing a good understanding of why people engage in a particular activity and its importance along a range of dimensions is therefore vital.

The second assumption is that communities are homogenous, composed of similarly endowed households with common characteristics (Waylen et al. 2013). It is therefore assumed that ALPs implemented at the community level will have widespread uptake and reach the resource users of interest. Yet there are social and political structures that control access to resources and opportunities at the community level (Béné et al. 2009). There is also substantial evidence that natural resource use differs according to the relative wealth of community members and that the poorest households in a community are often those most dependent on natural resources (Kümpel et al. 2010). However, dependence is not the same as use. In a recent study, 7978 households across 24 developing countries were surveyed, and the results showed that the use of biodiversity by the richest 20% of households was 5 times higher than that of the poorest 40% of households (Angelsen et al. 2014). It is therefore necessary to be clear about the overall objectives of an intervention. To be effective purely in terms of conservation outcomes, alternatives need to generate benefits for the right people (i.e., those most heavily exploiting the target resource). If, however, the primary aim is to compensate for the negative impacts of resource use restrictions on those most dependent on natural resources, then the alternative should target, or at least be accessible to, the most vulnerable members of a community. A detailed understanding of the ways in which natural resources are used by different sectors of society is therefore essential.

The third assumption is that targeting interventions at individuals will scale up to population‐level reductions in impact on the natural resources of conservation concern. This assumes the individual will influence a shift away from the environmentally damaging activity at the household level and shifts by individual households will then scale up to population‐level change. However, intrahousehold livelihood activities are dynamic. If one individual within the household is able to gain an income from an alternative activity, this may lead to a reallocation of labor and increased effort exploiting the target resource by another household member (Allison & Ellis 2001). Even if households do change their behavior, there are many exogenous factors that may undermine the conservation benefits of an intervention at the community and population levels. For example, in the Philippines, seaweed farming has been promoted as an alternative occupation for fishers, but Hill et al. (2012) showed that although some households did change from fishing to seaweed farming, the overall effect on fisher numbers was diluted by the growth in human population through births and in‐migration.

External stimuli, such as markets, are also highly influential at the community level and can even change the nature of the conservation threat. For example, the increase in the price of cocoa has encouraged many smallholder farmers in Cameroon to create or expand their cocoa farms. Although this has resulted in a shift from nontimber forest product harvesting to cocoa farming in certain areas, it has also resulted in increased degradation of high conservation value habitats (van Vliet 2010). It is therefore important to be mindful of the dynamic, multilevel nature of the social–ecological systems within which ALPs are implemented (Berkes 2007). Engendering change at one level may not necessarily scale up to result in the desired conservation outcome at a higher level and change is not guaranteed to be stable over time. Dialogue with individuals and groups at multiple levels can help in understanding the evolving nature of opportunities and threats from different perspectives so that management approaches can be adapted accordingly (Cundill et al. 2011).

The prevalence of inaccurate assumptions such as these highlights insufficient consideration of the complexities of livelihoods by conservation practitioners. Livelihood‐focused interventions that fail to recognize these complexities are likely to fail in achieving their conservation objectives.

## Understanding the Complexity of Livelihoods

Livelihood‐focused conservation interventions often mistakenly equate the wants and needs of local people with monetary benefits and economic substitutes (Berkes 2012). Focusing on livelihoods in monetary terms masks the complexity of rural livelihoods in developing countries. Just as the concept of poverty has been redefined as multidimensional (Davies et al. 2014), so too has the concept of livelihoods. To conceptualize the multiple influences on people's livelihood strategies, a number of sustainable livelihoods approaches (SLAs) have been developed since the 1990s. They tend to consist of a theoretical framework alongside a set of principles that guide livelihood analyses and subsequent interventions (Toner & Franks 2006). The most notable SLA was developed by the UK Department for International Development (Carney 1998). Following SLA principles, a livelihood can be defined as the living gained through the productive use of assets in activities to which access has been granted through social, institutional, and political processes. A livelihood is considered sustainable when it can withstand and recover from stresses and shocks and can maintain or enhance a household's assets while not undermining the natural resource base (Scoones 1998).

SLAs highlight the range of activities a household engages in as part of a dynamic livelihood strategy and draw attention to the fact that a portfolio of activities is likely to be needed if a household is to achieve its livelihood goals. There are multiple reasons for households to diversify their livelihood activities. Some are voluntary and proactive in response to new opportunities or as a means of reducing vulnerability by anticipating and ameliorating risks, others are necessary coping mechanisms resulting from deteriorating conditions or sudden shocks (Ellis 2000). Households in developing countries often lack access to insurance, so many prefer livelihood strategies that spread risk rather than maximize returns (Barrett et al. 2001). Engaging in a wide range of activities is one of the best ways of spreading risk because it allows households to change the mix and relative importance of activities depending on their circumstances at any point in time. In the context of uncertainty, permanently abandoning a particular livelihood activity and substituting it for a newly introduced activity would be considered risky. For example, evidence from Thailand, Nicaragua, and Tanzania suggests that if households are used to getting a small daily income from fishing, they are unwilling to abandon this activity to focus on activities such as aquaculture that require significant investment and can take months to generate revenue (Torell et al. 2010).

However, it cannot be assumed that all livelihood choices are made solely with the aim of achieving the optimal balance between material gains and risk. Attitudes to risk vary among individuals, and people engage in activities for a multitude of reasons, including enjoyment (Pollnac & Poggie 2008). Ultimately, what people do has meaning for them, and this should not be ignored (Gough et al. 2007).

In seeking to understand livelihood strategies, it is necessary to move beyond simply considering a household's current portfolio of livelihood activities and acknowledge that the livelihood trajectory of each household will be different. Some households will be “hanging in,” continuing with the same activities purely to maintain their current standard of living, whereas others will be “stepping up,” investing in and enhancing their current activities or “stepping out” by accumulating sufficient assets to launch into completely different activities (Dorward et al. 2009). The strategy of a household at any given time is determined by its goals and aspirations, stage in the demographic life cycle, assets, and the constraints imposed or opportunities provided by social and political structures (Niehof 2004; Gough et al. 2007). Households in the stepping out category are more likely to have the capacity and assets to mitigate the risks involved in moving from an environmentally damaging activity to an alternative. In contrast, those hanging in are likely to switch only at severe levels of resource depletion or if they are under substantial pressure due to resource access restrictions. In either case, this could lead to considerable hardship.

SLAs highlight that a single activity promoted by an ALP is unlikely to fully substitute for the range of tangible and intangible benefits provided by the destructive activity it was intended to replace. However, by adopting a SLA and acknowledging the diversified nature of livelihood strategies in developing countries, conservation practitioners can improve their understanding of the role and function of environmentally damaging behaviors within household livelihood portfolios. Exploring the range of activities conducted by different households according to their asset profiles can help in determining which households have the greatest environmental impact and those most vulnerable to conservation‐imposed resource access restrictions. Such information can help improve the targeting of future interventions. Finally, SLAs provide a framework for exploring the social–ecological system within which the livelihood strategies of households are embedded. This includes giving due consideration to endogenous and exogenous trends, as well as the power relations, politics, and institutions both within conservation programs and the broader landscape that determine the differential access to livelihood opportunities by different groups (de Haan & Zoomers 2005).

## Moving Forward by Realigning ALPs with the Current Conservation Agenda

One critique of ALPs describes them as “initiatives that promote unsustainable solutions that are poorly adapted to people's capacities, have limited market appeal and fail to reflect people's aspirations for their future” (IMM 2008). Here we have highlighted that poorly conceived projects result from shortcomings in conceptual design and inadequate understanding of the social context. Some conservation programs already give thorough consideration to the complexity of livelihoods (e.g., IMM 2008; FFI 2013), but outdated assumptions are still prevalent in conservation on the ground. It is therefore important to recognize the complexity inherent in intervening to alter people's livelihood strategies. For instance, even if a promoted activity is adopted, it may only provide a degree of substitution within the target population. For example, in Cameroon, cocoa farming appears to be shifting the focus of men away from forest‐based activities such as hunting, but only for a proportion of the year because the main income from cocoa is in October–November and the majority is spent over the festive period. Households are therefore still reliant on income from other activities, including hunting, to pay school fees the following September (van Vliet 2010).

As conservationists are increasingly reminded through international forums of their ethical responsibility to do no harm to local people, it may be more appropriate to target livelihood‐focused interventions at those most vulnerable to resource access restrictions as a form of compensation. A focus on enhancing the existing livelihood strategies of this group, by making livelihood activities more effective, more efficient, or lower risk, can also help make them more resilient to change (Torell et al. 2010). Livelihood‐focused interventions targeted at other groups should be designed carefully to ensure they address real, locally defined needs and lead to positive social outcomes, but it is unlikely that these interventions alone will suffice when it comes to achieving conservation outcomes. Experience from Uganda indicates that the real value of livelihood‐focused interventions from a conservation perspective is in improving local attitudes toward conservation, thus reducing conflict and increasing cooperation between resource users and protected area authorities (Blomley et al. 2010). Building good community relations through effective livelihood‐focused interventions that establish a clear link to conservation may be a more appropriate and realistic aim than using these interventions as a direct behavior‐change tool. Such interventions could be considered a form of incentive to collaborate with conservation.

Whichever approach is used, it is important not to lose sight of the broader context because it is often macrolevel processes, which are usually beyond the scope of livelihood‐focused interventions, that determine how livelihood pathways evolve. For instance, it was the international price of gold that eventually led to a switch from rattan harvesting to gold panning in North Sulawesi (Clayton et al. 2002). External trends may also ultimately offset the conservation gains of an intervention, even if local livelihood strategies do change as a result (Hill et al. 2012). The wider processes of social and ecological change must therefore be considered if livelihood‐focused interventions are to remain locally relevant and effective in conservation terms.

Finally, terminology is important. Shifting from the term *ALP* to the broader term *livelihood‐focused intervention* removes the key, and simplistic, assumption of substitution. We believe this shift will lead to more realistic and nuanced theories of change in project design and evaluation. This small terminological change would be a step toward working more holistically with local people to improve both their well‐being and the conservation status of the species and ecosystems upon which they depend.
